# Experimental realization of non-Abelian permutations in a three-state non-Hermitian system

**DOI:** 10.1093/nsr/nwac010

**Published:** 2022-01-18

**Authors:** Weiyuan Tang, Kun Ding, Guancong Ma

**Affiliations:** Department of Physics, Hong Kong Baptist University, Hong Kong, China; Department of Physics, State Key Laboratory of Surface Physics, and Key Laboratory of Micro and Nano Photonic Structures (Ministry of Education), Fudan University, Shanghai200438, China; Department of Physics, Hong Kong Baptist University, Hong Kong, China

**Keywords:** non-Abelian permutation, non-Hermitian physics, topological physics, acoustics

## Abstract

Eigenstates of a non-Hermitian system exist on complex Riemannian manifolds, with multiple sheets connecting at branch cuts and exceptional points (EPs). These eigenstates can evolve across different sheets—a process that naturally corresponds to state permutation. Here, we report the first experimental realization of non-Abelian permutations in a three-state non-Hermitian system. Our approach relies on the stroboscopic encircling of two different exceptional arcs (EAs), which are smooth trajectories of order-2 EPs appearing from the coalescence of two adjacent states. The non-Abelian characteristics are confirmed by encircling the EAs in opposite sequences. A total of five non-trivial permutations are experimentally realized, which together comprise a non-Abelian group. Our approach provides a reliable way of investigating non-Abelian state permutations and the related exotic winding effects in non-Hermitian systems.

## Teaser

Resting on the novel non-Hermitian topology, we experimentally demonstrate state permutations in a non-Hermitian system follow non-Abelian, or “order-dependent” exchange rules.

## INTRODUCTION

Permutation is a process of both fundamental and practical importance. For example, one way to distinguish fermions from bosons is to consider the exchange of the wave functions of two or more identical particles. Permutations of multiple states can emerge as the phenomenon of multi-state geometric phases [1,2]. They are generally non-commutative and can therefore be mapped to non-Abelian groups. This perspective suggests the possibility of emulating non-Abelian permutations by the parallel transport of three or more degenerate states. However, despite notable attempts in the fields of optics [3], cold atoms [4] and other topological systems [5,6], its realization remains a considerable experimental challenge, with the excitation and manipulation of multiple degenerate but coupled modes posing a major obstacle.

Recent advances in non-Hermitian physics have sparked the development of many intriguing applications related to optics and other classical waves [7,8]. Although non-Hermitian systems can be straightforwardly constructed from Hermitian systems by the inclusion of loss and/or gain or non-reciprocal hopping, they possess unique characteristics that are not found in their Hermitian counterparts. Perhaps the most notable distinction is that the eigenvalues are generally complex numbers. This simple fact permits the existence of multiple eigenvalue Riemann sheets connected at branch cuts [9–11]. The endpoints of the branch cuts are branch-point singularities known as exceptional points (EPs). Encircling an EP inevitably crosses one or multiple branch cuts—a process that causes the eigenstates to be exchanged and can even produce fractional winding numbers [11–17]. These fascinating behaviors, which are useful for topological energy transfer [11] and asymmetric mode switching [16] applications, have a topological origin: a non-Hermitian system lives on a complex Riemannian manifold that naturally permits state permutations. Hence, non-Hermitian systems offer a new vantage point for the study of state permutations. Recent theoretical investigations suggest that the encircling of multiple order-2 EPs or higher-order EPs is non-Abelian in character [13,18–21] and can give rise to a myriad of exotic winding effects [16,22–25]. These findings are consistent with the group theory point of view, as at least three degrees of freedom are required for non-Abelian processes to emerge. However, experimental confirmations of these proposals are lacking.

**Figure 1.**
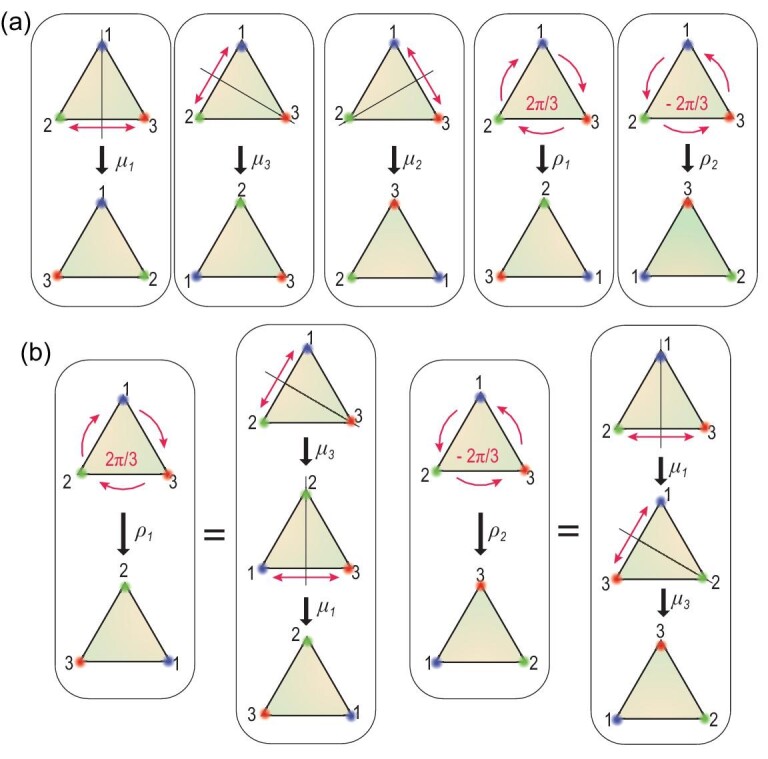
Operations comprising the ${D_3}$ group. (a) The five non-trivial operations of the non-Abelian ${D_3}$ group depicted as symmetry operations on an equilateral triangle. The ${\mu _{1,}}\!,$${\mu _2}$ and ${\mu _3}$ operations flip the triangle about the mirror axis that goes through Corners 1, 2 and 3, respectively. The ${\rho _1}$ and ${\rho _2}$ operations are the clockwise and anticlockwise rotations of $2\pi /3$ that permutate all three corners. (b) The ${\rho _1}$ and ${\rho _2}$ operations can be achieved by concatenating ${\mu _1}$ and ${\mu _3}$ in opposite sequences. The non-Abelian nature is clearly seen as ${\mu _1} \ \circ \ {\mu _3} \ne {\mu _3}\ \circ \ {\mu _1}$.

In this work, we theoretically investigate and experimentally realize the non-Abelian permutations of three states in a non-Hermitian system. By embedding the system's Riemannian manifolds in a 3D parameter space, two exceptional arcs (EAs), smooth trajectories of order-2 EPs, are found. As we will show, encircling them induces a unique permutation of the eigenstates. Five distinct types of state permutations are realized by encircling the EAs individually or sequentially. These five permutations, together with an identity element, holistically form a dihedral group of degree three, called the ${D_3}$ group, which can be used to describe the symmetry operations on an equilateral triangle, as shown in Fig. 1. All five permutations in Fig. 1a)and the equivalent permutations in Fig. 1b)are experimentally realized via a stroboscopic approach [17,23,26–28] in acoustic experiments. We further show that the permutation operations are described by $3 \times 3$ unitary matrices, also known as *U*(3) non-Abelian Berry phases (NABPs), which connect the three-state evolutions on the system's complex Riemannian manifold.

## RESULTS

### EAs in a three-state non-Hermitian system

We begin with an exceptional nexus (EX) that emerges in a three-state non-Hermitian Hamiltonian $H\ = \ ( {{\omega _0} + i{\gamma _0}} ){\boldsymbol{ I}} + {H_{E\!P}}$, where ${\omega _0} + i{\gamma _0}$ denotes the complex onsite energy and ${H_{E\!P}}$ determines the core physics and has the following form:

(1) \begin{eqnarray*} &&{ {H_{E\!P}}\! \left( {\eta ,\zeta ,\xi } \right) = \ } \\ &&\quad \kappa \left[ {\begin{array}{@{}*{3}{c}@{}} {\sqrt 2 \left( {i + \eta } \right)}&\quad 1&\quad 0\\ 1&\quad {i\zeta + \xi }&\quad 1 \\ 0&\quad 1&\quad { - \sqrt 2 \left( {i + \eta } \right)} \end{array}} \right]\\ &&\quad +\, i\sqrt 2 \kappa\! \left( {\begin{array}{@{}*{3}{c}@{}} g&\quad 0&\quad 0\\ 0&\quad 0&\quad 0\\ 0&\quad 0&\quad { - g} \end{array}} \right). \end{eqnarray*}

**Figure 2.**
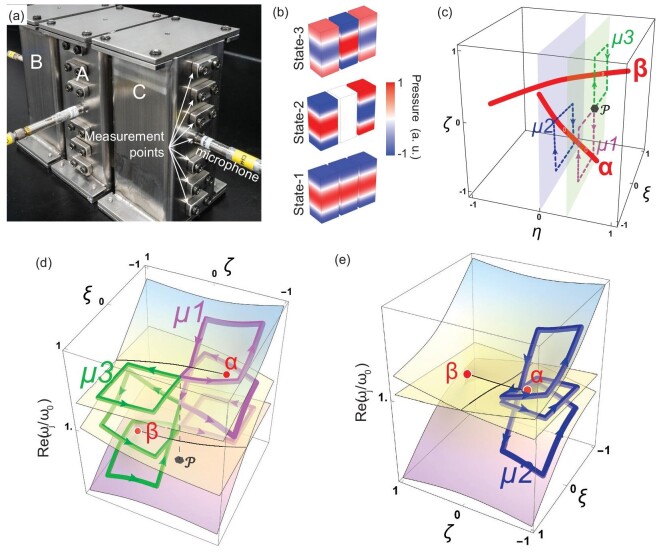
Three-state acoustic system and state permutations by encircling EAs. (a) The experimental set-up of the ternary coupled acoustic cavities. (b) The simulated acoustic modes in the absence of non-Hermicity $( {\eta \ = \ \zeta \ = \ \xi \ = \ g\ = \ 0} )$. (c) Two EAs (solid red curve) lie in a 3D parameter space spanned by $\eta \zeta \xi $. The evolutions along the purple, green and blue dashed loops produce the operations ${\mu _1}$, ${\mu _3}$ and ${\mu _2}$, respectively. (d–e) respectively show the eigenvalue Riemann surfaces on the $\zeta \xi $-plane at $\eta \ = \ 0.33$ (the light-green plane in c) and $\eta \ = \ 0$ (the light blue plane in (c)). The surfaces from the bottom to top correspond to States 1, 2 and 3 when non-Hermiticity is present. The red dots mark the intersection with EA-$\alpha $ and EA-$\beta $. The thin black curves are branch cuts, while the purple, green and blue routes indicate the evolutions of the eigenvalues along ${\mu _1}$, ${\mu _3}$ and ${\mu _2}$, respectively. All eigenvalues are normalized by the onsite resonant frequency ${\omega _0} = \ 19\,729\ {\rm{rad}}/{\rm{s}}$. The surface hues in (d–e) are for aesthetic purposes only and do not convey physical information.

${H_{E\!P}}$ lives on a 3D parameter space spanned by $( {\eta ,\ \zeta ,\ \xi } ) \in {\mathbb{R}^3}$. There is also another parameter *g*, which for the convenience of discussion is not regarded as a separate dimension. Here, all coefficients are normalized by $\kappa $ (where $\kappa < 0$), which is the hopping coefficient between neighboring sites. A ternary cavity system can be used to experimentally realize the Hamiltonian in acoustics, as shown in Fig. 2a. The second-order cavity mode is chosen as the onsite resonance mode. The parameters $\eta $ and $\xi $ represent detuning to onsite resonant frequencies, while $i\zeta $ and $ig$ are introduced as losses. Figure 2b)shows the three eigenmode profiles from a lower frequency (State 1) to a higher frequency (State 3) in the absence of non-Hermiticity. More details of the experimental set-up are given in Section III of the supplementary information.

When $g\ = \ 0$, an EX exists at $( {\eta ,\ \zeta ,\ \xi } ) = ( {0,0,0} )\ $, which is an order-3 EP that connects to four EAs [23], each of which is a trajectory of order-2 EPs formed by two of the three eigenstates of Equation (1). These three eigenstates constitute a Hilbert space, which can be figuratively referred to as a fiber, at each parametric point $( {\eta ,\ \xi ,\ \zeta } )$, thus forming fiber bundles that stick to the base manifold in the parameter space. The non-Hermiticity of the system means that the three eigenvalue Riemannian sheets connect at branch cuts, which naturally allows the exchange of states by encircling the EPs. Hence, each EA can be characterized by the two surrounding eigenstates in the permutation. As shown in Fig. 1, the permutations ${\mu _1}$ and ${\mu _3}$ constitute two generating operations of the ${D_3}$ group and the other elements of the ${D_3}$ group can be generated by ordered operations of ${\mu _1}$ and ${\mu _3}$, i.e. ${\rho _1} = {\mu _1}\ \circ {\mu _3}$, ${\rho _2} = {\mu _3}\ \circ \ {\mu _1}$ and ${\mu _2} = {\mu _3}\ \circ \ {\mu _1} \ \circ \ {\mu _3}$. The identity element is not of interest here, since it generates no changes. The issue of how to realize two EAs that possess the ${\mu _1}$ and ${\mu _3}$ types of permutation is therefore crucial for the demonstration of non-Abelian permutations.

In order to achieve this, we introduce the second term in Equation (1). When $g \ne 0$, the EX splits into two order-2 EPs in the $\zeta \xi $ plane at $\eta \ = \ 0$. In this way, the four EAs converging at the EX become a pair of smooth EAs. Figure 2c)shows the two EAs, denoted $\alpha $ and $\beta $, for $g\ = \ 0.61$. We note that the way in which the EAs connect is dependent on the sign of *g* (see Sections I and II of the supplementary information for details) and we focus on the case with positive values of *g* in the main text. This configuration allows us to trace the evolution of states around the EAs, making it suitable for analysing the non-Abelian permutation of states that is the focus of this work.

### Two generating permutations by encircling an EA

We first demonstrate two generating operations, ${\mu _1}$ and ${\mu _3}$, that exchange two of the three states. To facilitate the discussion, we order the eigenstates based on the real parts of the eigenfrequencies at the starting point of the loop. We set $\eta \ = \ 0.33$, which is depicted as a light-green plane in Fig. 2c. The $\zeta \xi $ plane intersects with both EAs at two EPs, as shown by the red dots on the eigenvalue Riemann surface (real part) in Fig. 2d. The purple loop encircles EA-$\alpha $, which is formed by the coalescence of States 2 and 3 at $\eta \ = \ 0.33$. Hence, one complete cycle must cross the branch cut once, resulting in the swapping of the two states, and consequently the operation ${\mu _1}:123 \to 132$ is realized. Likewise, it is straightforward to see that ${\mu _3}$, which encloses EA-$\beta $, exchanges States 1 and 2, i.e. ${\mu _3}:123 \to 213$.

**Figure 3.**
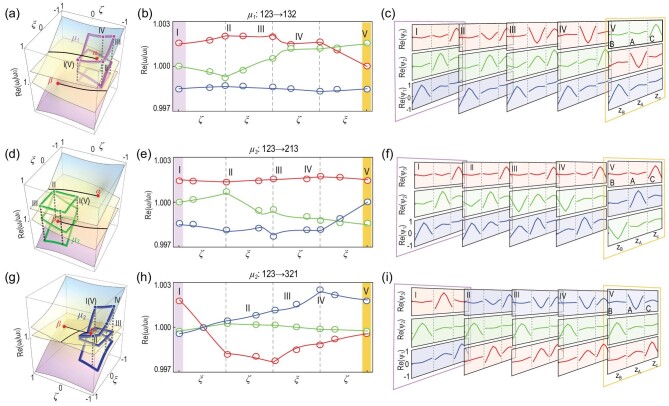
Two-state permutations. The three types of two-state permutations are represented by the eigenvalue Riemann surfaces (real parts) in (a) ${\mu _1}$, (d) ${\mu _3}$ and (g) ${\mu _2}$. The corresponding evolutions of the eigenvalues and the measured eigenfunctions are shown in (b, e, h) and (c, f, i), respectively. In (b, e) and (h), the markers and lines show the experimental and theoretical results, respectively. The eigenvalues and eigenfunctions of States 1, 2 and 3 are labeled in blue, green and red, respectively. The Roman letters indicate the selected parametric points, which are also labeled in (a, d) and (g) for better visualization of the encircling evolutions.

These permutations are experimentally observed via a stroboscopic approach. The parameters of the acoustic system are tuned to the specific values defined by the chosen loop. To achieve this, a Green's function method is used to determine the experimental parameters at each parametric point from the measured pressure response spectra (the details of this process are presented in Sections IV and IX of the supplementary information). The complex eigenfrequencies are then obtained by using the above parameters from the Green's function method and their real parts are plotted as the open circles in Fig. 3b)and e for ${\mu _1}$ and ${\mu _3}$, respectively. The solid lines in the figure show the theoretical results and their colors share the same notation as in Fig. 1. The measured eigenvalues are schematically labeled on the Riemann surfaces in Fig. 3a)and d, which clearly delineate the evolutions associated with ${\mu _1}$ and ${\mu _3}$. The salient feature that two states exchange at the branch cuts is clearly seen, and thus Fig. 3b)and e agree well with our expectation.

Next, the evolutions of eigenfunctions are also obtained experimentally by measuring the acoustic field profile in all three cavities (see Section V of the supplementary information for details). The results indeed show the swapping of eigenfunctions across the branch cut where the real parts of the eigenvalues cross. In Fig. 3c)and f, we plot the representative eigenfunctions at five chosen points along the encircling path and state exchanges are observed. The results shown in Fig. 3c)can be taken as an example. We see that at starting Point I, State 2 (shown in green) has a large amplitude at Site A (the middle site). As the system is driven along the ${\mu _1}$ path, the amplitude at Site A gradually diminishes, while that at Site C increases. At the last two points, State 2 at Point IV smoothly connects to State 3 at Point V, as a direct consequence of crossing the branch cut. Likewise, State 3 at Point IV (shown by the red lines) connects to State 2 at Point V. Meanwhile, State 1 remains almost unchanged throughout the evolution. Upon the completion of one closed cycle, the final outcome is the exchange of States 2 and 3.

We further remark that as the parameters change, the eigenfunctions of the three states also vary. It is therefore crucial to correctly identify how the eigenfunctions evolve along the parametric points, especially in the vicinity of the branch cut where the state exchange takes place. We examine the inner products for all the neighboring states, i.e. ${| {\langle \psi _{i, l + 1}^L|\psi _{j, l}^R\rangle } |^2}$, where $|\psi _{j, l}^R\rangle $ is the right eigenfunction of the *j*th state at the parametric point *l* and $\langle \psi _{i, l + 1}^L|$ is the left eigenfunction of the *i*th state at point $l + 1$, where $i,\ j\ = \ 1,\ 2,\ 3$. The two neighboring eigenfunctions that yield an inner product close to unity are connected by parallel transport [29]. This procedure was performed for all states at all the parametric points presented in our work.

The state permutation induced by ${\mu _1}$ can be captured by a $U( 3 )$ NABP [1] (see Section VI of the supplementary information for details). Using the eigenvectors of ${H_{E\!P}}$ as a basis, the NABP for ${\mu _1}$ is:

(2) \begin{equation*} {\rm{\ }}{{{\boldsymbol U}}_{{\mu _1}}} = \left( {\begin{array}{@{}*{3}{c}@{}} 1&\quad 0&\quad 0\\ 0&\quad 0&\quad 1\\ 0&\quad 1&\quad 0 \end{array}} \right)\ . \end{equation*}

From Equation (2), we can further obtain a multiband Berry phase as:

(3) \begin{equation*} {{\rm{\Theta }}_{{\mu _1}}} = \ - {\rm{Im }}\big[ {\ln \big( {\det {{\boldsymbol{U}}_{{\mu _1}}}} \big)} \big] = \ - \pi . \end{equation*}

This phase factor can be observed as a $\pi $-phase difference between State 2 at Points I (shown in green) and V (shown in red) in Fig. 3c. These results are consistent with the knowledge that an order-2 EP possesses a fractional winding number of 1/2 and the fact that encircling the EP twice restores both states with a Berry phase of $\pi $. The ${\mu _3}$-induced state permutation can also be seen by tracing the eigenfunction evolutions in Fig. 3f)and its corresponding NABP is:

(4) \begin{equation*} {\rm{\ }}{{{\boldsymbol U}}_{{\mu _3}}} = \left( {\begin{array}{@{}*{3}{c}@{}} 0&\quad 1&\quad 0\\ 1&\quad 0&\quad 0\\ 0&\quad 0&\quad 1 \end{array}} \right)\ , \end{equation*}

which also yields a Berry phase of ${{\rm{\Theta }}_{{\mu _3}}} = \ - \pi $ from Equation (3). Although ${{\rm{\Theta }}_{{\mu _1}}}$ and ${{\rm{\Theta }}_{{\mu _3}}}$ are the same, the two NABPs ${\boldsymbol{{U}}_{{\mu _1}}}$ and ${{\boldsymbol{U}}_{{\mu _3}}}$ are different and they do not commute.

### Non-Abelian permutations by sequentially encircling two EAs

As shown in Fig. 1a, the ${D_3}$ group has two elements that describe three-state permutations, denoted as ${\rho _1}:123 \to 231$ and ${\rho _2} = \ 123 \to 312$. These can be attained by concatenating ${\mu _1}$ and ${\mu _3}$ in different orders, i.e. ${\rho _1} = {\mu _1} \circ {\mu _3}:123 \to 213 \to 231$ and ${\rho _2} = {\mu _3} \circ {\mu _1}:123 \to 132 \to 312$, as shown in Fig. 1b. The permutation outcomes of ${\rho _1}$ and ${\rho _2}$ are clearly different and this is a manifestation of the non-Abelian characteristics, i.e. ${\mu _3} \circ {\mu _1} \ne {\mu _1} \circ {\mu _3}$.

**Figure 4.**
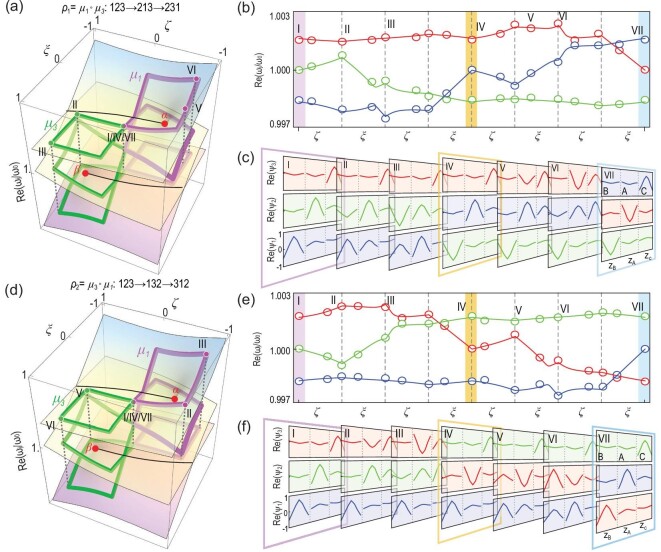
Three-state permutations and their non-Abelian characteristics. The two types of three-state permutations ${\rho _1}$ and ${\rho _2}$ are represented by their eigenvalue Riemann surfaces (real parts) in (a and d), respectively. (b and c) respectively show the measured evolutions of eigenvalues and eigenfunctions by encircling first EA-$\beta $ and then EA-$\alpha $, which corresponds to the operation ${\rho _1} = {\mu _1}\ \circ {\mu _3}$. (e and f) The measured evolutions of the eigenvalues and eigenfunctions realize ${\rho _2} = {\mu _3}\ \circ {\mu _1}$. The Roman letters indicate the selected parametric points, which are also labeled in (a and d) for better visualization of the encircling evolutions.

The three-state permutations are achieved by sequentially encircling both EA-$\alpha $ and EA-$\beta $. Without loss of generality, we can anchor the two loops ${\mu _1}$ and ${\mu _3}$ at a common vantage point $\mathcal{P}\,\ \eta ,\zeta ,\xi = ( {0.33,\ 0,\ 0} )$, as depicted by the black hexagon in Fig. 2c. The point $\mathcal{P}$ is also the starting and end point of the encircling. In Fig. 4a–c, the ${\mu _3}$ operation is executed first by encircling EA-$\beta $, which swaps States 1 and 2. The ${\mu _1}$ operation is then carried out by encircling EA-$\alpha $, thus exchanging the new States 2 and 3. The net result is the swapping of all three states, as defined by ${\rho _1}$. The ${\rho _2}$ operation is also experimentally achieved by first encircling $\alpha $ and then $\beta $, as shown in Fig. 4d–f. The two experimental outcomes, i.e. the mode profiles at the parametric Point VII in Fig. 4c)and f, are clearly distinct, thus unambiguously validating the non-Abelian characteristics. Again, we can summarize the three-state permutations with the NABPs as:

(5) \begin{eqnarray*} {{{\boldsymbol U}}_{{\rho _1}}} &=& {{\boldsymbol{U}}_{{\mu _1}}}{\boldsymbol{ }}{{\boldsymbol{U}}_{{\mu _3}}} = \left( {\begin{array}{@{}*{3}{c}@{}} 0&\quad 1&\quad 0\\ 0&\quad 0&\quad 1\\ 1&\quad 0&\quad 0 \end{array}} \right)\! , \\ {{\boldsymbol{U}}_{{\rho _2}}} & =& {{\boldsymbol{U}}_{{\mu _3}}}{\boldsymbol{ }}{{\boldsymbol{U}}_{{\mu _1}}} = \left( {\begin{array}{@{}*{3}{c}@{}} 0&\quad 0&\quad 1\\ 1&\quad 0&\quad 0\\ 0&\quad 1&\quad 0 \end{array}} \right)\! . \end{eqnarray*}

It is clear that ${{\boldsymbol{U}}_{{\rho _1}}} \ne {{\boldsymbol{U}}_{{\rho _2}}}$, although the Berry phases in both cases are ${{\rm{\Theta }}_{{\rho _1}}} = {{\rm{\Theta }}_{{\rho _2}}}\ = \ 0$ [${\rm{mod}}( {2\pi } )$], as can be identified from the mode profiles at the parametric Points I and VII in Fig. 4c)and f. This verifies the non-Abelian character by encircling different types of EAs.

### Multiple permutations of encircling EAs

We have already demonstrated four of the five non-trivial permutations depicted in Fig. 1a. The remaining operation is ${\mu _2}:123 \to 321$, which exchanges States 1 and 3. Following the rule used to label the EAs, we would expect that EPs exist near to the EX that correspond to the permutation ${\mu _2}$. This can be attained by shifting the encircling loop of EA-$\alpha $ to $\eta \ = \ 0$, as shown in Fig. 2c. At first sight, it seems counterintuitive that ${\mu _2}$ can exist in our system, since the hopping between Sites B and C is zero in Equation (1). To see how ${\mu _2}$ emerges, we first note that $\eta $ represents onsite detuning in Sites B and C, thus letting${\rm{\ }}\eta $ cross zero causes the inversion of the lowest and highest frequency modes (States 1 and 3). At $\eta \ = \ 0$, the two order-2 EPs (EP-$\alpha $ and EP-$\beta $ in Fig. 2e) are linked by a branch cut that is parallel to the $\zeta $ axis, which connects the lowest and highest frequency sheets. Hence, an evolution that follows the blue loop in Fig. 2e)exchanges States 1 and 3 and leaves State 2 unchanged, thus realizing ${\mu _2}$.

The ${\mu _2}$ operation is also experimentally realized using our acoustic system. The results for the eigenvalues and eigenfunctions are shown in Fig. 3h)and i, respectively, where the exchange of States 1 and 3 can clearly be seen. We have also computed the corresponding NABP:

(6) \begin{equation*} {{\boldsymbol{U}}_{{\mu _2}}} = \left( {\begin{array}{@{}*{3}{c}@{}} 0&\quad 0&\quad 1\\ 0&\quad 1&\quad 0\\ 1&\quad 0&\quad 0 \end{array}} \right)\ , \end{equation*}

and ${{\rm{\Theta }}_{{\mu _2}}} = \ - \pi $. We further remark that, as an element in ${D_3}$, ${\mu _2} = {\mu _3} \circ {\mu _1} \circ {\mu _3}$ (or ${\mu _2} = {\mu _1} \circ {\mu _3} \circ {\mu _1}$). This indicates that the permutation ${\mu _2}$ can be treated as the operation of encircling the EAs multiple times in our non-Hermitian system. To show this, we can shift the position of the blue loop in Fig. 2c)slightly to $\eta \ = \ 0.055$, so that it transverses three different branch cuts, with each traversal exchanging two states. These results are presented in Section VII of the supplementary information. Since ${\mu _2}$ completes the ${D_3}$ group here, all other operations that encircle the EAs in Fig. 2c)multiple times must be equivalent to the single operation shown in Figs 3 and 4.

## DISCUSSION AND CONCLUSION

A common practice for characterizing topological manifolds is to consider equivalence classes of loops, in which winding numbers play a vital role. Non-Hermitian topology can be characterized by the eigenvalue winding number, sometimes called the eigenvalue vorticity or discriminant number [28,30,31], which is often considered to be sufficient to reveal the topological structure of the complex Riemann surfaces. However, our results show that eigenvalues are not directly associated with state permutations. Even the eigenvector winding numbers underlain by the Berry phase ${\rm{\Theta }}$ do not contain explicit information on state permutations. The state permutations and their non-Abelian characteristics are disclosed either by tracing the parallel transport of all three states or by computing the NABP matrix. Hence, the EAs and their interactions constitute the non-Hermitian counterparts of the knot and link structures of nodal lines in Hermitian band structures [19,20,32].

A question naturally arises as to how the winding numbers relate to the non-Abelian permutations demonstrated in this work. To illustrate this, we recall that the two processes defined by ${\rho _1}$ and ${\rho _2}$ yield identical Berry phases ${{\rm{\Theta }}_{{\rho _1}}} = {{\rm{\Theta }}_{{\rho _2}}}\ = \ 0$ [${\rm{mod}}( {2\pi } )$], which can be regarded as the same eigenvector winding number. We have numerically confirmed that the eigenvalue winding numbers for ${\rho _1}$ and ${\rho _2}$ are also identical in these two cases and are consistent with their Berry phases. As discussed above, the evolutions ${\rho _1}$ and ${\rho _2}$ are equivalent to performing both ${\mu _1}$ and ${\mu _3}$ in opposite orders. However, the two concatenated loops ${\mu _1}$ and ${\mu _3}$ are equivalent to the larger loop encircling both EA-$\alpha $ and EA-$\beta $ (see Section VIII of the supplementary information). When this loop is followed, three complete cycles are needed to restore all three states, which gives rise to a fractional winding number of 2/3 [23,33]. In other words, one complete parametric cycle following ${\rho _1}$ and ${\rho _2}$ does not recover all the states. It follows that the states after one cycle are dependent on the states at the starting point. This is the reason for the non-Abelian outcomes demonstrated in our work.

In summary, we have successfully demonstrated that all the non-trivial operations comprising the ${D_3}$ group can be realized by encircling EAs in a three-state non-Hermitian system. Our work builds on recent developments in non-Hermitian physics that have introduced a kaleidoscope of EP structures with distinct topological characteristics. Experimentally, our studies are based on the stroboscopic approach so that the non-adiabatic transitions typically encountered in dynamic evolutions can be avoided [16,34–36]. Our work and the methodology can be extended to study knot and link structures formed by different EAs [37–40]. The combined strength of these theoretical developments and experimental techniques in non-Hermitian physics, in conjunction with the rich arsenal of non-Abelian theories, will open new avenues to the discovery of exotic phenomena and the development of rich applications in a diversity of fields. For example, non-Abelian permutations around multiple EAs provide additional degrees of freedom to manipulate wave propagation [16] and on-chip energy transfer [11]. Relating to our work are several recent studies proposing a new class of anyonic-parity-time symmetric systems [41,42] that can benefit applications such as lasers [43]. On the other hand, the existence and evolutions of multiple EPs in a multi-parameter phase space give rise to rich opportunities of more sophisticated usage of EPs, which may benefit applications such as sensors [44,45], absorbers [46,47], scattering control [48], etc.

## DATA AVAILABILITY

Data are available upon request to the corresponding authors.

## Supplementary Material

nwac010_Supplemental_File
