# Supplementary material for: Experimental realization of non-Abelian permutations in a three-state non-Hermitian system
Source: Natl Sci Rev. 2022 Jan 18;9(11):nwac010. doi: 10.1093/nsr/nwac010 (PMC9746695; doi:10.1093/nsr/nwac010)
Supplement: nwac010_Supplemental_File [file nwac010_supplemental_file.pdf]

Supplementary Information

"Experimental Realization of Non-Abelian Permutations in a Three-State Non-Hermitian System"

Weiyuan Tang[1], Kun Ding[2], Guancong Ma[1]

[1]Department of Physics, Hong Kong Baptist University, Kowloon Tong, Hong Kong, China

[2]Department of Physics, State Key Laboratory of Surface Physics, and Key Laboratory of Micro and Nano Photonic Structures (Ministry of Education), Fudan University, Shanghai 200438, China

This Supplementary Information contains 9 sections, 6 figures, 3 tables, and 7 references.

CONTENTS

I. The exceptional arcs
II. The EAs and the exceptional nexus
III. Experimental setup
IV. The acquisition of eigenvalues and other system parameters from experimental data
V. The acquisition of eigenfunctions from experimental data
VI. The non-Abelian Berry phase matrix
VII. The permutation of state-1 and 3
VIII. The equivalence loop of the concatenated loops
IX. Parameters retrieved from the measurements

I. The exceptional arcs

Here we mathematically show the existence and forms of the exceptional arcs (EAs). We begin with Eq. (1) in the main text, reproduced here

$$H_{EP}(\eta,\zeta,\xi)=\kappa\begin{bmatrix}\sqrt{2}(i+\eta) & 1 & 0\\ 1 & i\zeta+\xi & 1\\ 0 & 1 & -\sqrt{2}(i+\eta)\end{bmatrix}+i\sqrt{2}\kappa\begin{pmatrix}g & 0 & 0\\ 0 & 0 & 0\\ 0 & 0 & -g\end{pmatrix}, \quad (1)$$

$\kappa,\eta,\zeta,\xi,g\in\mathbb{R}$. Here we set $\kappa=-1$, then the characteristic polynomial $p=\det(\omega\mathbf{I}-H_{EP})$ is

$$p(\omega)=\omega^3+\omega^2(\xi+i\zeta)-2\omega(\eta+ig)[\eta+i(2+g)]-2(\xi+i\zeta)[\eta+i(1+g)]^2. \quad (2)$$

For convenience, we define

$$a_3=1, a_2=(\xi+i\zeta), a_1=-2(\eta+ig)[\eta+i(2+g)],$$
$$a_0=-2(\xi+i\zeta)[\eta+i(1+g)]^2. \quad (3)$$

So that Eq. (2) becomes

$$p(\omega)=a_3\omega^3+a_2\omega^2+a_1\omega+a_0. \quad (4)$$

Differentiate $p(\omega)$ with respect to ω produces

$$q(\omega)=b_2\omega^2+b_1\omega+b_0, \quad (5)$$

where $b_2=3a_3, b_2=2a_2, b_0=a_1$. Then, the discriminant Δ of the characteristic polynomial p is

$$\Delta(p)=\prod_{i<j}(\omega_i-\omega_j)^2=(-1)^{N(N-1)/2}\det[\mathrm{Syl}(p,q)], \quad (6)$$

where $N=3$ for our system, and $\mathrm{Syl}(p,q)$ is the Sylvester matrix of the polynomials p and q,

$$\mathrm{Syl}(p,q)=\begin{pmatrix}a_3 & a_2 & a_1 & a_0 & 0\\ 0 & a_3 & a_2 & a_1 & a_0\\ b_2 & b_1 & b_0 & 0 & 0\\ 0 & b_2 & b_1 & b_0 & 0\\ 0 & 0 & b_2 & b_1 & b_0\end{pmatrix}. \quad (7)$$

Since we only focus on the region in which all four parameters are smaller than 1, we retain the terms up to the third order

$$\Delta(p)\approx-72\xi^2\zeta-144\xi\eta\zeta-27\xi^2+27\zeta^2+192\eta^2g+72\zeta^2g-64g^3+i(72\xi^2\eta-64\eta^3-144\xi\zeta g-72\zeta^2\eta-54\xi\zeta+192\eta g^2). \quad (8)$$

At the exceptional points, both the real and imaginary parts of the discriminant $\Delta(p)$ are nil, namely

$$\mathrm{Re}[\Delta(p)]=0,\ \mathrm{Im}[\Delta(p)]=0. \quad (9)$$

In the main text, $g=0.61$ for the EAs in Fig. 2(c), Eq. (9) gives

$$-72\xi^2\zeta-144\xi\eta\zeta-27\xi^2+27\zeta^2+177.12\eta^2+70.92\zeta^2-14.53=0, \quad (10)$$
$$-64\eta^3+72\xi^2\eta-72\zeta^2\eta-195.84\xi\zeta+71.44\eta=0. \quad (11)$$

Equations (10) and (11) give two sets of curvilinear surfaces in $\eta\zeta\xi$ space that intersect in the formation of the EAs, as depicted in Fig. S1.

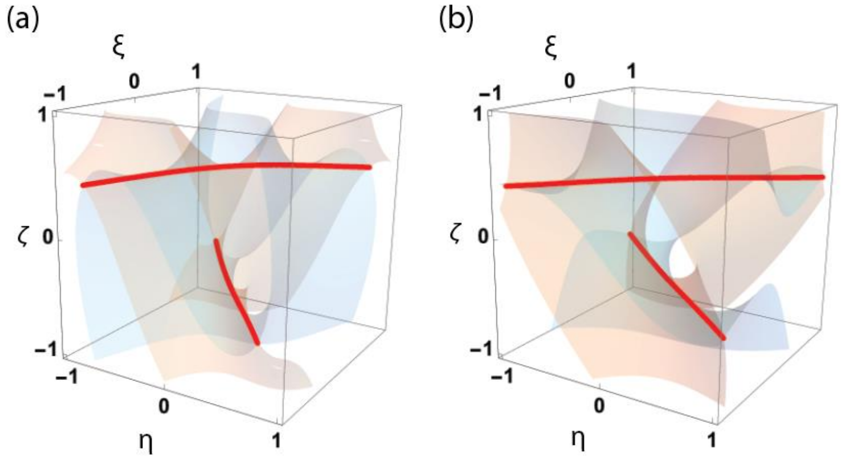

Figure S1. (a) The accurate EAs with $g = 0.61$. The orange and blue surfaces correspond to $\mathrm{Re}[\Delta(p)] = 0$ and $\mathrm{Im}[\Delta(p)] = 0$, respectively, and their intersections are highlighted in red, corresponding to the EAs. (b) The EAs obtained by the approximated equations (10) and (11).

II. The EAs and the exceptional nexus

The non-Hermitian Hamiltonian, namely Eq. (1) in the main text, is derived from the model used in Ref. [1], which can produce an "exceptional nexus" (EX), i.e., an order-3 EP at which all order-2 EAs form the cusp singularities. When $g = 0$, the EX appears at $\eta, \zeta, \xi = 0$, as shown in Fig. S2(b). Two cusp singularities are perpendicular to each other. Figures S2(a) and S2(c) show the behavior of these EAs when $g < 0$ and $g > 0$, respectively. We see that the EX disappears, and the two cusp singularities formed by the EAs become two smooth EAs. Such configurations are convenient for our investigation of state permutations, since they can be easily achieved and monitored by encircling one EA at a time. Comparing Figs. S2(a) and (c), we can see that the EAs for the $g < 0$ and $g > 0$ cases connect in different manners. When $g < 0$, each EA corresponds to a definite state permutation, but it is not the case when $g > 0$. The state permutation along the EAs can change when $g > 0$, and thus provide the possibility to realize the various permutations in the D_3 group.

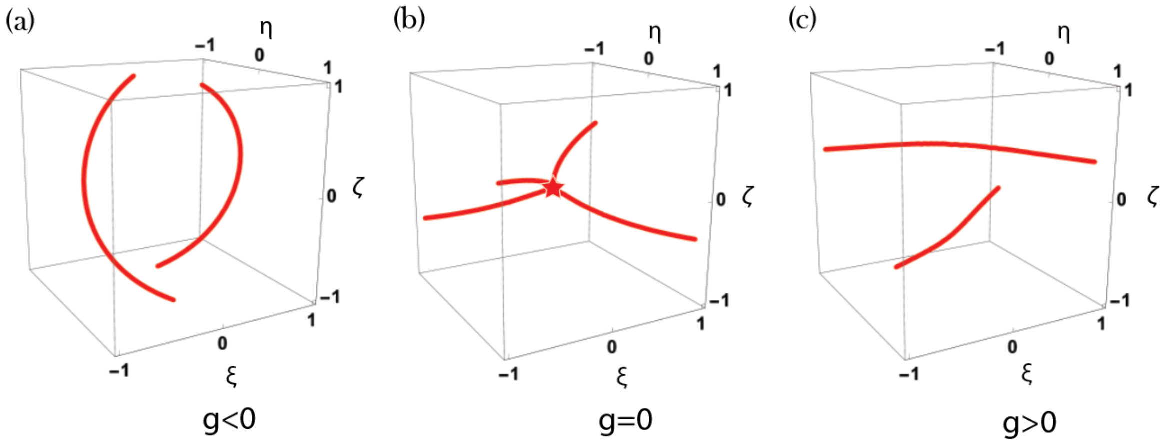

Figure S2. The EAs for (a) $g < 0$, (b) $g = 0$, and (c) $g > 0$. When $g = 0$, the EAs kiss at $\eta, \zeta, \xi = 0$ in the formation of an EX, which is an order-3 EP (the red star).

III. Experimental setup

We use three cuboid acoustic-cavity resonators to realize the non-Hermitian Hamiltonian, as shown in Fig. 2(a) of the main text. The stainless-steel cavities are filled with air and have a height $h = 110$ mm and a square cross-section with a side length of 44 mm. The cavities are joined together by small horizontal holes with a cross-sectional area of 17 mm^2, which introduces a hopping of $\kappa = -49.5$ rad/s. The second-order mode, which has a cosine acoustic profile with two nodal planes, is employed to realize the onsite orbital. The Hermitian eigenmodes, that is, with the absence of differential loss and gain, are depicted in Fig. 2(b) in the main text. A small port with a radius of 2 mm is opened on the top of each cavity for the external excitation, and a loudspeaker is used to pump at cavity-B. These ports also introduce additional radiative loss, which contributes to γ_0 in our model.

The realization of state permutations requires the additional loss and frequency offset to be precisely controlled by the relevant acoustic parameters in our experiment. In our experiments, the additional loss is achieved by placing small pieces of acoustic sponge at the bottom of specific cavities. The frequency offset is achieved by tuning the volume of the acoustic cavity, which is implemented by inserting a specific amount of putty. We have experimentally characterized the effects of sponge and putty, as shown in Fig. S3(a) and (b), respectively. The loss and detuning are determined by fitting the spectral responses of a single cavity using the Green's function.

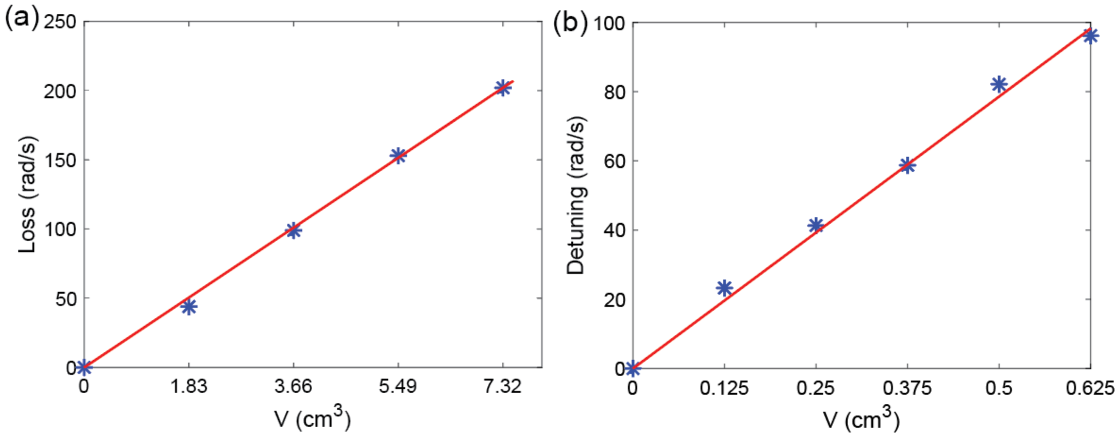

Figure S3. (a) The linear relationship between loss and the volume of the acoustic sponge. (b) The linear relationship between detuning and the volume of putty. The red curves are the linear fit, and the blue markers represent extracted data from measurements.

IV. The acquisition of eigenvalues and other system parameters from experimental data

The eigenvalues of our system can be acquired from the measured pressure response spectra through the Green's function method[1–4]. The Green's function can be expanded in the left and right eigenvectors $\left|\psi_j^R(\lambda_l)\right\rangle$ and $\left\langle\psi_j^L(\lambda_l)\right|$ with $j = 1, 2, 3$ labeling the states and λ_l denoting the parametric coordinate of the l-th step along a closed loop

$$\overleftrightarrow{G}(\omega, \lambda_l) = \sum_{j=1}^{3} \frac{\left|\psi_j^R(\lambda_l)\right\rangle\left\langle\psi_j^L(\lambda_l)\right|}{\omega-\omega_j(\lambda_l)}. \tag{12}$$

Here $\omega_j(\lambda_l)$ is the eigenvalues. The pressure responses measured at a specific parametric step λ_l inside the three coupled cavities are

$$P(\omega, \lambda_l) = \left\langle m\middle|\overleftrightarrow{G}(\omega, \lambda_l)\middle|s\right\rangle, \tag{13}$$

wherein $|s\rangle$ and $|m\rangle$ are 3×1 column vectors denoting the source and probe positions. In our experiment, the source is placed on the top of cavity A and three identical microphones pick up the pressure response at all three cavities. Therefore, $|s\rangle = (0 \quad 1 \quad 0)^T$ and $|m\rangle = (1 \quad 0 \quad 0)^T$, $(0 \quad 1 \quad 0)^T$ and $(0 \quad 0 \quad 1)^T$ for the probing at cavity B, A, and C, respectively. The measured data at the three cavities are then fitted against Eq. (13) by a genetic-algorithm-assisted least-square fitting. All the parameters $\omega_0, \gamma_0, \kappa, \eta, \zeta, \xi, g$, together with the eigenvalues ω_j, are thus obtained.

V. The acquisition of eigenfunctions from experimental data

The permutations of states are observed by tracing the eigenfunctions of the three states as they evolve along different loops. Hence the acquisition of eigenfunctions is a crucial step. For a three-state non-Hermitian system, the eigenfunctions can be constructed by the onsite modes

$$\left|\psi_j^R(\lambda_l)\right\rangle = \begin{bmatrix} a_{j,A}(\lambda_l)|\varphi_A\rangle \\ a_{j,B}(\lambda_l)|\varphi_B\rangle \\ a_{j,C}(\lambda_l)|\varphi_C\rangle \end{bmatrix}, \tag{14}$$

$$\left\langle\psi_j^L(\lambda_l)\right| = [b_{j,A}(\lambda_l)\langle\varphi_A|, \quad b_{j,B}(\lambda_l)\langle\varphi_B|, \quad b_{j,C}(\lambda_l)\langle\varphi_C|]. \tag{15}$$

Here, $\left|\varphi_{A,B,C}\right\rangle$ is the isolated onsite mode of the individual cavity A, B, and C. Its real-space representation is a 7×1 column vector since there are seven measurement positions on each cavity. Thus, in the real-space representation, $\left|\psi_j^R(\lambda_l)\right\rangle$ is a 21×1 column vector and $\left\langle\psi_j^L(\lambda_l)\right|$ is a 1×21 row vector. The real parts of the 21 elements of $\left|\psi_j^R(\lambda_l)\right\rangle$ are the results shown in Fig. 3(c, f, i) and Fig. 4(c, f) in the main text.

To obtain those results, the first step is to obtain $\left|\varphi_{A,B,C}\right\rangle$ by the Green's function

method mentioned before

$$P_B(\omega) = \frac{\langle m|\varphi_B\rangle\langle\varphi_B|s\rangle}{\omega-[\omega_0+i\gamma_0+i\sqrt{2}\kappa(1+g+\eta)]}, \tag{16}$$

$$P_A(\omega) = \frac{\langle m|\varphi_A\rangle\langle\varphi_A|s\rangle}{\omega-[\omega_0+i\gamma_0+\kappa(i\zeta+\xi)]}, \tag{17}$$

$$P_C(\omega) = \frac{\langle m|\varphi_C\rangle\langle\varphi_C|s\rangle}{\omega-[\omega_0+i\gamma_0-i\sqrt{2}\kappa(1+g+\eta)]}, \tag{18}$$

wherein $|m\rangle$ and $|s\rangle$ now become 7×1 column vectors. $|s\rangle$ only has one nonzero element. The retrieved parameters are used in Eqs. (16-18). The data to be fitted are the measured pressure responses at 31 frequencies near ω_0 at 7 positions on each isolated cavity.

The second step is to obtain the coefficients $a_{j;A,B,C}, b_{j;A,B,C}$. This is done by fitting the pressure responses of the three coupled cavities measured at totally 21 positions (7 for each cavity) at the same 31 frequencies

$$P_j(\omega,\lambda_l) = \langle m|\overleftrightarrow{G}(\omega,\lambda_l)|s\rangle = \sum_{j=1}^{3}\frac{\langle m|\psi_j^R(\lambda_l)\rangle\langle\psi_j^L(\lambda_l)|s\rangle}{\omega-\omega_j}. \tag{19}$$

Here, $|m\rangle$ and $|s\rangle$ are 21×1 column vectors and there is also only one nonzero element in $|s\rangle$. Upon attainment of the coefficients $a_{j;A,B,C}, b_{j;A,B,C}$, the right and left eigenfunctions $|\psi_j^R(\lambda_l)\rangle$ and $\langle\psi_j^L(\lambda_l)|$ are readily obtained. This procedure is repeated for each parametric step $\lambda_l = (\eta,\zeta,\xi)$ along the designated loops.

Because the non-Hermitian system lives on a self-intersecting complex Riemannian manifold, special care must be taken to correctly identify the evolution of eigenstates. First, the parallel transport of states must be satisfied. Our experimental raw data, which are obtained using a stroboscopic approach, inevitably carry arbitrary phases that are caused by the acoustic excitation at each parameter point. The arbitrary phases are extracted as $\theta_j(\lambda_{l+1}) = \mathrm{Im}\left[\ln\langle\psi_j^L(\lambda_l)|\psi_j^R(\lambda_{l+1})\rangle\right]$, and then compensated at each step[4,5]. This way ensures that the eigenfunctions at neighboring steps satisfy the parallel transport under a constant $U(1)$ gauge, which is the phase factor at the initial step $\theta_j(\lambda_1)$. Second, to obtain the correct connection of states at neighboring parametric points, the inner products $|\langle\psi_j^L(\lambda_l)|\psi_j^R(\lambda_{l+1})\rangle|$ are computed for all states at all steps as an indicator. This procedure is necessary to identify state exchanges. In addition, the eigenvectors are intrinsically mixed due to the presence of non-Hermicity, meaning that the eigenfunction profiles do not stay the same during the encircling process. The evolution of states is therefore correctly enforced by comparing the inner products at each step.

VI. The non-Abelian Berry phase matrix

The evolutions of states around one or multiple EPs, including their permutations, can be captured by the non-Abelian Berry phase matrix. For a single band, the Berry phase is a $\boldsymbol{U}(1)$ connection between the eigenstate at different parametric locations in an adiabatic evolution. The formalism can be generalized for a multiband system, in which case the Berry phase becomes a $\boldsymbol{U}(n)$ matrix, wherein n is the number of consecutive bands under consideration [6]. For our system, $n = 3$ so that

$$\left|\psi_j^R(\lambda_{\mathcal{L}})\right\rangle = \sum_{k=1}^{n=3} U_{jk}|\psi_k^R(\lambda_1)\rangle, \tag{20}$$

with U_{jk} is an element in

$$\widetilde{\boldsymbol{U}} = \prod_{l=1}^{\mathcal{L}-1} \boldsymbol{M}(\lambda_l, \lambda_{l+1}), \tag{21}$$

wherein $M_{jk}(\lambda_l, \lambda_{l+1}) = \left\langle \psi_j^L(\lambda_l) \middle| \psi_k^R(\lambda_{l+1}) \right\rangle$. $\widetilde{\boldsymbol{U}}$ is a unitary matrix, but in general, it does not take the forms of $\boldsymbol{U}$ as shown in Eqs. (2, 4, 5, 6) in the main text. To obtain those specific results, the state vectors at the starting point needs to be prepared as $\left|\overline{\psi_1^R}(\lambda_1)\right\rangle = (1 \quad 0 \quad 0)^T$, $\left|\overline{\psi_2^R}(\lambda_1)\right\rangle = (0 \quad 1 \quad 0)^T$, $\left|\overline{\psi_3^R}(\lambda_1)\right\rangle = (0 \quad 0 \quad 1)^T$. (For these three state vectors to be valid, the encircling path must be sufficiently distant from the EP, otherwise the eigenvectors become skewed. This condition is always met in our calculations and experiments, since we do not approach the EPs.) Although it is difficult to actually prepare these state vectors in stroboscopic experiments, they are connected to the eigenvectors by a unitary transformation on the eigenvectors $\left|\overline{\psi_j^R}(\lambda_1)\right\rangle = \boldsymbol{P}\left|\psi_j^R(\lambda_1)\right\rangle$, wherein $\boldsymbol{P}$ is given by $\widetilde{H}_{EP3} = \boldsymbol{P}^\dagger H_{EP3} \boldsymbol{P}$ such that $\widetilde{H}_{EP3}(\lambda_1)\left|\overline{\psi_j^R}(\lambda_1)\right\rangle = \omega_j(\lambda_1)\left|\overline{\psi_j^R}(\lambda_1)\right\rangle$. By applying the same transformation $\boldsymbol{P}$ to the eigenvectors $\left|\psi_j^R(\lambda_l)\right\rangle$ (and $\left\langle\overline{\psi_j^L}(\lambda_l)\right| = \boldsymbol{P}^\dagger\left\langle\psi_j^L(\lambda_l)\right|$) for the subsequent steps λ_l, we can then obtain the $\boldsymbol{U}$ shown in the main text. It is easy to see that $\boldsymbol{U}$ and $\widetilde{\boldsymbol{U}}$ are connected by the same transformation $\boldsymbol{U} = \boldsymbol{P}^\dagger \widetilde{\boldsymbol{U}} \boldsymbol{P}$.

When the path is a closed loop, i.e., $\lambda_{\mathcal{L}} = \lambda_1$, $\boldsymbol{U}$ is gauge-invariant. We can further obtain a phase factor, sometimes also called a multiband Berry phase

$$\Theta = -\mathrm{Im}[\ln(\det \boldsymbol{U})]. \tag{22}$$

We remark that the phase factor Θ given by Eq. (22) is identical to the result obtained by tracing the cyclic evolution of a single eigenstate along an EP-encircling loop multiple times until the recovery of all states. The latter method was used to obtain the non-Hermitian Berry phase, such as in Refs. [1,2,5].

VII. The permutation of state-1 and 3

In the main text, we show that state-1 and 3 can exchange by encircling an EA in the $\zeta\xi$

plane at $\eta = 0$, which generates the μ_2 operation. By referring to the Cayley table of the D_3 group, it is easy to see that $\mu_2 = \mu_3 \circ \mu_1 \circ \mu_3$, which is graphically shown in Fig. S4(a). This process can be found in our system. By setting $\eta = 0.055$, i.e., slightly shifting the light-blue plane and the blue loop in Fig. 2(c) in the main text, the evolution delineates the path shown in Fig. S4(b). The state exchanges take place sequentially, as shown in Fig. S4(c). By shifting back to $\eta = 0$, the three exchanges occur at the same point, which are the results shown in Fig. 3(g-i) in the main text.

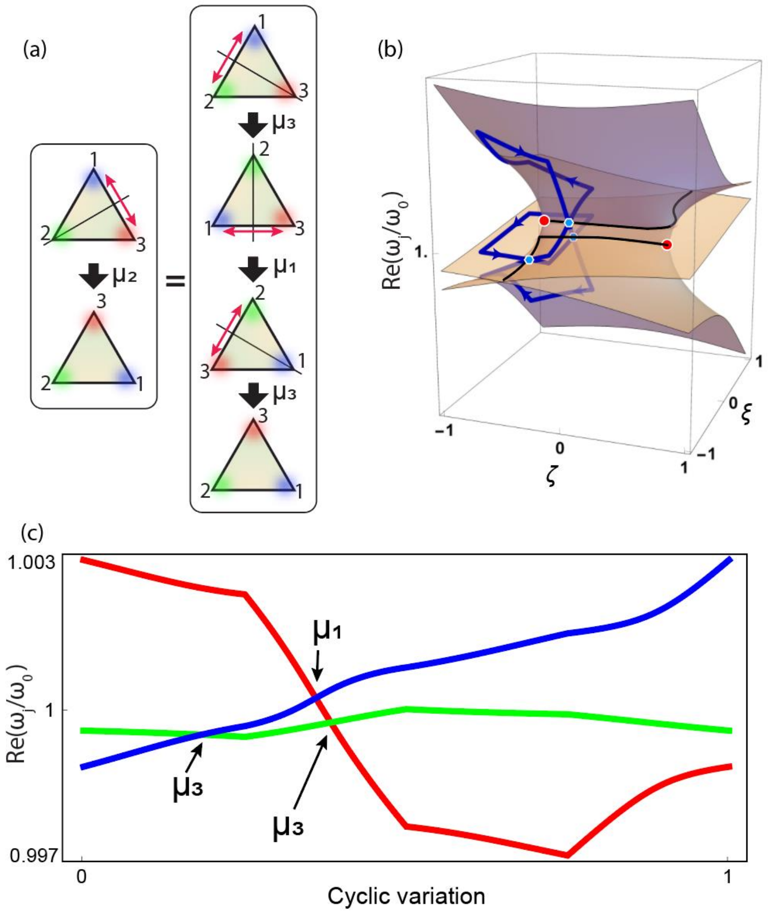

Figure S4. (a) The μ_2 operation can be generated by $\mu_2 = \mu_3 \circ \mu_1 \circ \mu_3$. (b) The eigenvalue Riemann surfaces near a pair of EPs (red dots) on the $\zeta\xi$-plane with $\eta = 0.055$. The branch cuts are depicted in black. The blue loop traverses the branch cuts three times and the intersecting points are marked by the blue dots. (c) Unwrapping the evolution, we can clearly identify the composition of $\mu_3 \circ \mu_1 \circ \mu_3$.

VIII. The equivalence loop of the concatenated loops

The operations ρ_1 or ρ_2 are generated by executing μ_1 and μ_3 in different sequences. The concatenated loops are equivalent to a single loop encircling both EA-α and β, as shown in Fig. S5(a). Following this loop, the evolution traverses all three sheets of the Riemannian surface [Fig. S5(b)]. In this case, three complete cycles are needed to recover all the eigenstates [1,7].

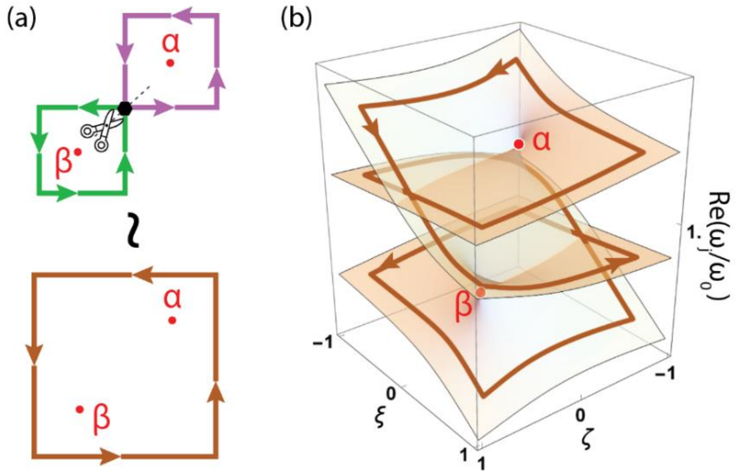

Figure S5. (a) The equivalence of the two loops each encircling one EA (also see Fig. 2c, main text) and a big loop that encircles both EAs. (b) The eigenvalue (real parts) Riemann surface shows the encircling of both EA-α and β within one loop.

IX. Parameters retrieved from the measurements

Here, we present the parameters in our experiments. The second longitudinal mode resonates at $f_0 = 3140\ \mathrm{Hz}$ so that $\omega_0 = 19729\ \mathrm{rad/s}$. The intrinsic loss of each cavity is $\gamma_0 = 83.5\ \mathrm{rad/s}$. The parametric points along the ρ_1 loop (which includes μ_1 and μ_3), the ρ_2 loop and the μ_2 loop are given in Tables S1–S3 accordingly. All these parameters are obtained using the Green's function method as described in Supplementary Information, Section III. To show the validity of our fitting method, we also represent some of the fitting results in Fig. S6.

Table S1. The parameters for the ρ_1 loop at $\eta = 0.33$.

Point #	ζ	ξ
1 (I)	0.00	0.00
2	0.16	0.00
3 (II)	0.54	0.00
4	0.54	0.35
5 (III)	0.54	0.51
6	0.16	0.51
7	0.00	0.50
8	0.00	0.30
9 (IV)	0.00	0.00
10	-0.40	0.00
11	-0.60	0.00
12(V)	-0.60	-0.16

13(VI)	-0.60	-0.44
14	-0.36	-0.41
15	0.00	-0.46
16	0.00	-0.26
17(VII)	0.00	-0.00

Table S2. The parameters for the ρ_2 loop at $\eta = 0.33$.

Point #	ζ	ξ
1 (I)	0.00	0.00
2	-0.40	0.00
3 (II)	-0.60	0.00
4	-0.60	-0.16
5 (III)	-0.60	-0.44
6	-0.37	-0.42
7	0.00	-0.43
8	0.00	-0.27
9 (IV)	0.00	0.00
10	0.16	0.00
11(V)	0.55	0.00
12	0.55	0.29
13(VI)	0.55	0.51
14	0.16	0.50
15	0.00	0.50
16	0.00	0.33
17(VII)	0.00	0.00

Table S3. The parameters for μ_2 loop $\eta = 0$.

Point #	ζ	ξ
1 (I)	-0.22	-0.46
2	-0.20	0.00
3	-0.21	0.44
4 (II)	-0.57	0.40
5	-0.79	0.40
6 (III)	-0.79	0.00
7 (IV)	-0.81	-0.44
8	-0.61	-0.45
9 (V)	-0.22	-0.46

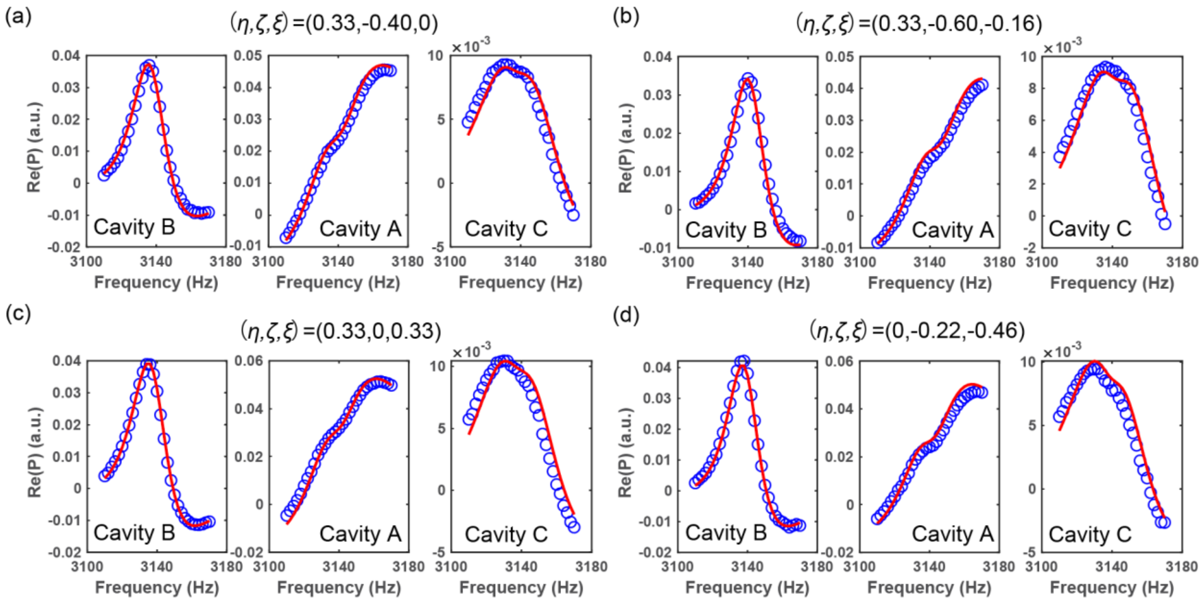

Figure S6. Selected results of measured pressure response spectra and fitting results. The blue markers are experimentally measured data. The red curves are fitted by using the Green's function method. Excellent agreement is seen.

REFERENCES

1. Tang W, Jiang X, Ding K *et al.* Exceptional nexus with a hybrid topological invariant. *Science* 2020;**370**:1077–80.

2. Ding K, Ma G, Xiao M *et al.* Emergence, Coalescence, and Topological Properties of Multiple Exceptional Points and Their Experimental Realization. *Phys Rev X* 2016;**6**:021007.

3. Ding K, Ma G, Zhang ZQ *et al.* Experimental Demonstration of an Anisotropic Exceptional Point. *Phys Rev Lett* 2018;**121**:085702.

4. Tang W, Ding K, Ma G. Direct Measurement of Topological Properties of an Exceptional Parabola. *Phys Rev Lett* 2021;**127**:034301.

5. Lee S-Y, Ryu J-W, Kim SW *et al.* Geometric phase around multiple exceptional points. *Phys Rev A* 2012;**85**:064103.

6. Vanderbilt D. *Berry Phases in Electronic Structure Theory: Electric Polarization, Orbital Magnetization and Topological Insulators*. Cambridge University Press, 2018.

7. Demange G, Graefe E-M. Signatures of three coalescing eigenfunctions. *J Phys Math Theor* 2012;**45**:025303.
